# Challenges faced by caregivers of children on antiretroviral therapy at Mutale Municipality selected healthcare facilities, Vhembe District, Limpopo Province

**DOI:** 10.4102/curationis.v40i1.1541

**Published:** 2017-08-28

**Authors:** Rhudzani V. Mafune, Rachel T. Lebese, Livhuwani H. Nemathaga

**Affiliations:** 1 Department of Public Health, University of Venda, South Africa; 2 Department of Advanced Nursing, School of Health, University of Venda, South Africa

## Abstract

**Background:**

Children depend solely on caregivers who can be either parents or guardians for drug administration to enhance adherence to antiretroviral treatment (ART), which might pose any number of challenges.

**Purpose:**

The purpose of this study was to explore and describe the challenges faced by caregivers of children on ART at Mutale Municipality, Vhembe District, Limpopo Province.

**Research design and method:**

The research design was qualitative, explorative, descriptive and contextual in nature. The population consisted of 16 caregivers who were 18 years of age and above, and mentally capable, irrespective of educational qualifications, caring for children aged between 0 and 15 years who were on ART between April 2013 and October 2014. Non-probability, purposive sampling was used to select the 16 caregivers. Required permission, approval and ethical clearance were obtained from the University of Venda Higher Degree Committee, Limpopo Provincial Health Department and relevant institutions. An in-depth, individual, unstructured interview method was used to collect data. One central question was asked: ‘What are the challenges you experience when caring for a child on antiretroviral treatment?’ Subsequent questions were based on the participants’ responses to the central question. Qualitative data were analysed by means of Tesch’s open-coding method.

**Results:**

The findings of this study revealed that participants, that is, caregivers of children on ART, experienced financial burdens because of transport costs needed to comply with follow-up dates and insufficient of money for food, clothing the child in need of care, pocket money for lunch boxes during school hours and time lost while waiting for consultations. Participants reported some level of stigmatisation against children on ART by family members, especially the husbands or in-laws of the secondary caregivers. Many primary and secondary caregivers seemed to have given up seeking support from government and community structures.

**Conclusion:**

The conclusions drawn from this research are that caregivers hardly receive any support from family members or the community. Fear of disclosing the HIV-positive status of children resulted in the delay of financial support from the government, thus leading to serious financial burden on the caregivers.

## Introduction and background

Antiretroviral (ARV) drug administration requires strict adherence to avoid the development of medication resistance, which would render the drugs ineffective and subsequently lead to morbidity and mortality (Brackis-Cott et al. 2003; Dlamini et al. 2009). ARV administration to children depends on caregivers to enhance adherence, which poses a challenge most of the time (Anabwani, Woldetsadik & Kline 2005). ARV drugs require frequent dosing and are mostly supplied in formulations that may be difficult for children to tolerate, such as large pills, bitter-tasting liquids and gritty powders (Mellins et al. 2002). The literature indicates that adherence can be affected by the relationship between the caregiver and the affected child. Children receiving therapy from foster parents are more compliant than those receiving drugs from biological parents or other relatives (Giacomet et al. 2003).

A study in Limpopo Province at the University of Venda showed that caregivers (mothers, aunts and grandmothers) indicated that fear of stigmatisation and prejudice made them delay seeking HIV-related healthcare for their children (Ahosi et al. 2014). Delay in accessing healthcare services is influenced by poverty, distance and availability of healthcare workers; stigma attached to HIV and AIDS has contributed to the separation of those suffering from infection from other members of the community (Health Systems Trust 2008). A study conducted by Nachega et al. (2012) identified that there was a misconception relating to use of kitchen utensils as it is said that these utensils should not be shared with HIV-positive individuals for fear of infection. It is these stigmatising attitudes that have prompted some caregivers to keep their children’s sickness a secret and sometimes ignore symptoms or treatment (Demmer 2011). Caregivers often experience extended grieving, psychological trauma, fears, anxiety of neglect and discrimination from family, peers and neighbours (Zhao et al. 2009). They were also reluctant to transfer knowledge of HIV status to the next line of caregiver and the truth, therefore, only came out when the child’s mother died which is also a challenge to the next caregiver (Venkataramani et al. 2009).

In response to these challenges, the HIV/AIDS and STI Strategic Plan for SA (2007–2011) addresses special needs of women and children by providing appropriate packages of services that include wellness, opportunistic infections management, ARV drugs and nutrition to children and adolescents who are HIV-positive or exposed. In sub-Saharan Africa, a number of non-governmental organisations were reported to be providing families with infected children with food aid, to minimise income and food security pressures on sick children and their caregivers. There is also the provision of the child support grant (CSG), disability grant and social grant to alleviate some of the poverty-related challenges, which are barriers to the adherence to ARV drugs (Skovdal et al. 2011). The Vhembe District in Limpopo Province has also developed a strategy for participating in greenery projects to provide infected people with vegetables. The focus of this article is to explore and describe the challenges faced by caregivers of children on antiretroviral treatment (ART) at Mutale Municipality, Vhembe District, Limpopo Province.

## Problem statement

The researcher is a primary healthcare nurse working in one of the primary healthcare facilities in Vhembe District. During her interaction with caregivers administering ARV drugs to children, she identified that caregivers experienced several problems in supervising treatment administration to children. The challenges experienced had an effect on adherence to treatment, which often resulted in the development of ARV resistance and concomitant increases in morbidity and mortality. Most of the caregivers are uneducated and elderly, which also contributes to poor treatment administration and adherence. It is against this background that the researcher sought to answer the following: ‘What are the challenges that are experienced by caregivers when caring for a child on ARV treatment?’

## Research purpose

The research purpose was to explore and describe the challenges faced by caregivers of children on ART at Mutale Municipality, Vhembe District, Limpopo Province.

## Research objectives

The research objectives were to explore and describe challenges faced by caregivers of children on ART.

## Significance of the study

The identified challenges might serve as a basis upon which programmes to improve the quality of life of caregivers of children on ART will be developed. Stakeholders may use the identified challenges to advocate for revision of existing policies or development of new policies to address the challenges experienced by caregivers. The government may use the identified challenges to establish more household-centred HIV services that take into account caregivers’ needs for support. The social development officials may use the identified challenges to strengthen HIV social support services.

## Research method and design

### Research design

The study followed a qualitative, explorative, descriptive and contextual design. Descriptive research was chosen because of its ability to enable the researcher to collect data in the field at the site where participants experience the issue or problem under study. Observations and interviews were used to assist the researcher in learning the meaning that the participants hold about the problem or issue.

### Population and sampling

The population included all caregivers who were 18 years of age and older, caring for children on ART aged 0 to 15 years, and receiving medications at Mutale Health Centre and its adjacent six clinics (Creswell 2007). The sample size of 16 caregivers was determined by data saturation. The researcher used her own judgement to select the aforementioned caregivers so that she could purposefully describe the challenges they experience in their healthcare roles. The researcher consulted the following records to select children’s age: paper ARV registers for the clinics and an electronic ARV register for the health centre, and then compiled a list. That was done after obtaining permission from the health centre managers. Standardised clinical records were consulted to identify their next consultation dates. The researcher signed for all patient folders on retrieval and when submitting them to the data capturers. The children’s ages were selected because it was during this period that children on ARV required constant supervision. The patient folders were reviewed in order to locate the appropriate sample.

### Data collection

In-depth, individual, unstructured interviews were conducted with all caregivers. Observations on non-verbal cues were made, an audiotape was used to capture verbal communication, and field notes were also taken. One central question was asked in English and also translated into Tshivenda which was: ‘What are the challenges you experience when caring for a child on ARV treatment?’ Translation was done by a Tshivhenda language expert. The researcher used probing and follow-up questions to stimulate participants’ thoughts and enhance their responses. Probing questions such as ‘tell me more about that’, reflecting on what the participant said, were asked to improve clarity.

### Data analysis

Tesch’s open-coding method was used to analyse data. Data were first transcribed verbatim in Tshivhenda, and then translated to English by a language expert. The researcher thereafter carefully read through the entire field notes (observations) and made interpretations as they came to mind in order to get a sense of the entire set of notes. Similar information was clustered and themes were developed. An independent coder was used to ensure appropriateness of the themes formed (Creswell 2007). Data saturation was reached after interviewing 16 caregivers when no new information was coming forth from the caregivers.

### Trustworthiness

Qualitative researchers need to assess whether their findings reflect the true state of human experience. Therefore, four criteria for establishing the trustworthiness of qualitative data were applied, namely credibility, transferability, dependability and confirmability (Lincoln & Guba 1999). The researcher ensured credible data and interpretations by spending sufficient time which was about 45–50 min with the participants and interviewing them more than once, verifying if the participants were still responding in the same way at a later stage. Engagement periods with the participants were prolonged to test for misinformation, distortions and to build trust with participants. The researcher went back to the field three times per participant to verify the information, ensuring prolonged engagement (interview, data verification and discussion of themes). Multiple methods of data collection such as interviews, observations and taking of field notes were used to address the research problem. Transferability was ensured by using the purposive sampling technique to select the participants who fulfilled the criteria described. An in-depth, individual, unstructured interview was conducted with each primary and secondary caregiver of children aged 0–15 years on ART at Mutale Municipality and each interview lasted between 45 min and 60 min. The researcher provided a thick description of the methodology used.

### Ethical consideration

A proposal has been presented to the School of Health Sciences’s Higher Degree Committee, the University Higher Degrees Committee, as well as the University’s Ethics Committee, to obtain ethical clearance (SHS/14/PH/03/1605). Privacy, confidentiality and anonymity were maintained (Burns & Grove 2005). Permission for approval of the study was requested from and granted by the Department of Health Provincial Research Committee, Vhembe District (Mutale Municipality). The researcher also ensured that the interviews were conducted in one of the consulting rooms in the health facilities to maintain privacy. The researcher maintained privacy through anonymity and confidentiality. The researcher ensured that the names and addresses of the participants did not appear anywhere in the study and participants were informed accordingly. Participants were provided with a code number. All the participants had an opportunity to sign an informed consent in English version and Tshivenda version. The researcher thus assured that the participants were provided with the information required in relation to the study. The researcher kept information in a locked cupboard, which only she could access or retrieve for data analysis, and the records were destroyed at the end of the study.

## Discussion of results

Data were collected from 16 caregivers at the selected primary health facilities of Mutale Municipality. The size of the sample was determined by data saturation. The participants’ ages ranged from 25 to 84 years and those of the children ranged from 0 to 15 years. All children were on CSG. Common sources of income were either a monthly CSG of approximately R360 or an old-age pension (OAP) of about R1200 per month. Each interview lasted between 45 min and 60 min. Interviews were conducted in one of the consulting rooms within the health facilities.

The following six subthemes were derived from the analysed data: (1) Financial challenges; (2) Treatment management challenges; (3) Disclosure and non-disclosure challenges; (4) Negative attitudes from family members and the community (stigma attached to HIV, ART and children); (5) Learning disabilities; (6) Inadequate support services from government and community structures.

### Subtheme one: Financial challenges

The majority of participants mentioned financial burdens that they experienced. The burden was said to be emanating from transport costs during follow-up visits and insufficient money for food, clothing for the child in need of care, pocket money for lunch boxes during school hours or time lost while waiting for consultations. On the other hand, caregivers indicated that they were allowed to collect ARV drugs in the absence of children in their care, which was said to be less expensive. Participants mentioned that ARV treatments were dispensed according to the children’s weight; therefore, they needed to accompany the children for monthly follow-ups. This study revealed that caring for children on ARV medication often resulted in caregivers borrowing money, accumulating and living in debt, footing or hiking for lifts to get access to the health facilities for follow-up. Some of the caregivers also pointed out that they did not have access to the CSG for those children, as it was controlled by the eldest child who does not prioritise the needs of the child who is on ARV medication. In addition, caregivers indicated that sometimes the money that is left behind by parents could only be accessed at the age of 18 years, which left the children with nothing to support them. Often, children would be stealing money from others and neighbours, which was evident in the comments:

‘I feel pain when I am unable to meet the basic needs of the child because he is an orphan. He might think that I do not want to give him money, whereas I do not have. Child is stealing money from other children and neighbours. Child is on child support grant. His elder brothers do not give him financial support; they say they are investing his child support grant in the bank for his education.’ (Caregiver 006, secondary caregiver, 35 years old, unemployed)

Many primary caregivers were uneducated and their opportunities for formal employment were limited; therefore, they depended on CSG. Caregivers indicated that they faced poverty and debt because the majority of them were unemployed, and others were dismissed from work because of their suspected HIV-positive status; their sources of income for survival were CSG, widows’ pension funds and OAP:

‘I borrow money from people to buy food, money for transport costs when accompanying the child for follow-up and pay back from my old-age pension of about R1350.’ (Caregiver 012, 65 years old, pensioner receiving pension grant)

‘Caring for a child who is on ARV is my secret. I was fired from work after eleven years of employment. I assumed that my employer seems to have discovered my secret. Nobody is helping me; I even spend seven days without food, washing clothes with leaves soap (museto). I have accompanied my child here by lift sometimes coming here is on foot.’ (Caregiver 005, 45 years old, unmarried, unemployed)

‘Child is on CSG and another source of income is my mother’s old-age pension. At first I did not experience problems … I mean challenges. Transport cost challenges started when we are expected to take children to the clinic every month for weight control since treatment is given according to the child’s weight. Initially, we were allowed to collect a two-month’s treatment supply in the absence of children, we were expected to visit clinics four times per year and I think it was reasonable.’ (Caregiver 022, 40 years old, unemployed, living on her mother’s old-age pension and CSG)

The results of this study are supported by the findings of the study conducted by Bejane, Havenga and Aswegen (2013) who also identified that elderly caregivers were using their OAP to meet the financial needs of their grandchildren. OAP was said to be the sole source of financial support for most caregivers. The older women’s unemployment status caused them to live in poverty and to depend on social grants to care for a number of children in their households (SANAC 2007). However, the study conducted by Kimani-Murage et al. (2010) found the instituted CSG does to a certain extent address the problem of food insecurity and was regarded by participants as being adequate in caring for children. From the observations made, there is thus a dire need for creative and effective income-generating strategies for caregivers of HIV-positive children; often they are themselves infected, hence they have limited opportunities for formal employment (Kompaore 2004). There is also a need for criteria for accessing social assistance grants (Hardy & Richter 2006; Kimani-Murage et al. 2010).

Considering the above, it is clear that the financial challenges can negatively impact on caregivers meeting the basic needs of the whole family and thus HIV-positive children in their care. This is in line with the study that was conducted in Agincourt, rural SA, which showed that because many mothers who were caregivers and were also infected, their opportunities for formal employment were limited; they depended on CSG. Many caregivers received support from family members, whereas few had access to special support and services for people living with HIV and AIDS (PLWHA), such as food supplements or support groups, which were related to the stigma associated with HIV, hindering caregivers from disclosing the child’s status outside the family and inhibiting access to special external support (Venkataramani et al. 2009).

### Subtheme two: Treatment management challenges

The study results indicate that caregivers were not given clear instructions on how to administer ARV drugs to children. It was also mentioned that children were coming home late and the frequency of taking medications was no longer considered, especially the evening doses. Caregivers also alluded to the fact that no proper instructions were given to them by health workers on how to administer treatment, and health workers were said to be always busy. The advanced age of the caregivers and their low literacy level was said to be contributing to poor understanding, which always leads to incorrect ARV administration.

‘Initially, I did not know how to give medications to the child because health workers did not explain to me thoroughly as he had a lot of patients to attend. I took the child to the clinic the other day when he was not feeling well and I was asked on how I have to give him his medication, it is then that I was told that I must feed him first and give him medications one hour after feeds; this means that I was not giving medication properly for a long time … esh!!!!’ (Caregiver 024, 68 years old, pensioner on old-age grant)

‘If I can go somewhere where I can be delayed to come back, the child can miss the doses for a period of two to three days. Then I blame myself and feel guilty if the child misses his doses because he will suffer from body weakness which will be relieved by giving a child water to drink. This also affects my socialising with relatives and friends, hence I feel isolated.’ (Caregiver 005, 45 years old, unmarried, unemployed)

‘The child was starting to come back home late; therefore, he was not following the frequency of taking medications, especially the evening doses. This affected his health and he went to the clinic several times and the nurses were not happy about it … I tell you.’ (Caregiver 006, secondary caregiver, 35 years old, unemployed)

It is also clear that caregivers were not opening up to talk about the nature of the disease and the consequences of these children not adhering to treatment. This was revealed when caregivers mentioned that they were using different strategies to deceive children so that they can take treatments as prescribed, which sometimes were in vain.

‘He has asked me the reason for taking medication, but I did not disclose. I have tried to tell the child that he is sick, because he did not want to take his medication. While he wanted to know about the condition he is suffering from, I told him that he is not growing well, unhealthy, does not have weight and short stature, and therefore the pills will make him grow well.’ (Caregiver 006, secondary caregiver, 35 years old, unemployed)

‘Sometimes he runs away from taking pills. I know that he is running away from taking pills because they are so big (said in a soft voice). At the time of taking medication, I watch him pouring water in a glass and directly observe him while swallowing pills I feel sorry for him.’ (Caregiver 021, 75 years old, pensioner depending on old-age pension and CSG)

Children who run away at the time of taking medications were forced to take treatments and they pretended as if they were drinking their pills meanwhile they were hiding the pills to be thrown away when the caregiver leaves:

‘He ran away at the time of taking medications. I used to chase him and he will come back and pretend as if he is drinking his pills meanwhile he was hiding the pills to be taken behind the curtains. I beat him with a lash to force him to take treatment.’ (Caregiver 006, secondary caregiver, 35 years old, unemployed)

ARV drugs were seen as a gift from God. Religious beliefs and practices facilitated adherence to medication (Watt et al. 2009). In contrast to this belief, a study conducted in Zimbabwe among faith-based organisations identified that the Apostolic faith religion believe that their prayers protect their children from all sorts of problems and, therefore, may not encourage children to continue with medication (Maguranga et al. 2012). Despite the increased availability of ARV drugs, children remain a neglected population (Sibhatu et al. 2011). It has been reported in another study that when children refuse to take medication, caregivers resort to beating them to force adherence (Paranthaman et al. 2009). The majority of caregivers reported that they did not explain to the children why they were taking them to the health facility or reasons for the reported visits to treatment centres (Kajubi et al. 2014). Oberdorfer et al. (2006) demonstrated that half of the caregivers who intended to disclose the child’s diagnosis in future expressed the need to be assisted by healthcare providers during disclosure. It is, therefore, concluded from the study findings that knowledge of one’s HIV status may affect ARV compliance and influence children’s participation in healthcare decision-making.

Good adherence is better achieved if patients stick to their ARV regimen for 95% of time (Attaran 2007) because it minimises resistance to affordable first-line drugs (Barth et al. 2011). ARV medications generally require frequent dosing and are supplied in formulations that may be difficult for children to tolerate (e.g. large pills, bitter tasting liquids and gritty powders), and these findings concur with the study results. Adults may remind the child to take his or her medicine, check medications to determine whether the child is taking them, which can instil a sense of self-responsibility, but this cannot be achieved if the child has no knowledge about his or her condition (Mellins et al. 2002). This is also in agreement with the study by Paranthaman et al. (2009) who have identified factors that influence adherence, such as side-effects caused by ARV drugs, size of the tablets and taste of the medicine and tablet regimens. Some children had to be forced to take their ARV medications regularly, causing frustrations for the caregivers. The caregivers explained that it was important for them to supervise the children to prevent children from throwing away ARV drugs which was observed by caregivers in this study (Bejane et al. 2013).

### Subtheme three: Disclosure and non-disclosure challenges

From the study findings, it is clear that there were reasons and experiences that made caregivers decide to disclose or to hide the nature of the disease and treatment, such as age of the child and the attitudes of family members or the community, whereas others did not know how to tell. It is also evident that both disclosure and non-disclosure can negatively or positively impact on the psychological aspect of both caregivers and children. It has been reported that caregivers who have disclosed the nature of the disease and treatment had contributed to the rejection and isolation of the children under their care. It was also stated that caregivers who did not disclose the nature of the disease or treatment suffered guilt feelings of hiding the status and treatment from the child and family members. Lack of family and community support, severe depletion of financial resources leading to unpaid accounts and development of concealment strategies when a child asks about the nature of the disease and treatment were mentioned as some of the challenges experienced by caregivers.

Caregivers displayed fear of opening up and to freely talk about the disease and treatment. This study identified that some participants were compelled to disclose the HIV-positive status and ART because some children were not adhering to ARV medications. The majority of caregivers disclosed an HIV-positive status because children were persistently enquiring about the duration and reasons for taking medications. The majority of caregivers said that they suffered from guilt of hiding an HIV-positive status from family members and friends in fear of mockery, verbal abuse, gossip, rejection at home and stigmatisation. Participants mentioned that children who were on ART were rejected, isolated, their kitchen utensils and basins were even separated from others by family members:

‘After disclosing to my husband and the child’s grandparents that the child is on ARV medication, they did not show any acceptance. They rejected the child and started to separate whatever the child came into contact with, for example, cups and even cutlery; it was a hurting situation. Since I took this child after the death of the mother who is my sister, family members said the child must relocate back to his own home so that he must suffer with his elder brothers and sisters; I was seriously hurt by those words.’ (Caregiver 006, secondary caregiver, 35 years old, unemployed)

In this study, the majority of caregivers withheld specific information in disclosing an HIV-positive diagnosis, whereas some caregivers offered an inaccurate explanation of the illness or false names of HIV diagnosis.

‘The child does not know the indication of taking treatment. He asked me and I did not tell. His elder sister once said it is time for him to go for follow-up ARV bloods; the child became inquisitive and needed a thorough explanation on ARV bloods. The child was no longer happy. He has asked me about the ARV, I said I don’t know them and what he is suffering from is confidential and I also have a condition that is confidential.’ (Caregiver 007, 70 years old, depending on CSG and old-age pension fund)

The majority of caregivers were secretly giving ARV to the children, whereas some caregivers shared their secret with close relatives or their children.

‘Ah, (pause). Ehh, ahh, ee, if he can ask me the nature of the disease or treatment, eee, this word cannot be uttered to children, ARV are unknown to children. I will tell him that the mother was suffering and on pills for the incurable disease that is all over the world, at the end I will say AIDS.’ (Caregiver 020, 75 years old, depending on OAP and CSG, widowed)

Participants have reported that if children insist on being informed about the nature of diseases and their treatments, they will ask health professionals to provide professional explanation—more especially where the child has tested HIV-positive and became aware of the results.

‘He became ill, was tested and found to be HIV-positive when his parents were no longer alive. He has asked me the reason for taking treatment and I have told him that it is for child health, to facilitate blood flow, he was checked and found to be having an incurable disease of the blood sugar diseases. (Laughing) I was lying. If he can ask continuously, I will go to the health professionals for assistance with disclosure.’ (Caregiver 021, HBC, 43 years old, who took the child on behalf of the immobile grandmother)

The literature corroborates the findings of this study in that it is believed that people think that being HIV-positive has to do with something in the person’s personal behaviour which contributed to the individual contracting the disease. Caregivers were said to be faced with unmet needs for support and guidance from health services regarding disclosure to the children. Some were even reluctant to inform their extended family members for fear of isolation (Paranthaman et al. 2009).

In agreement with the findings of this study, parents are said to delay or avoid disclosure of an HIV-positive status or ART to children because they believe that they do not know how to disclose properly (Kennedy et al. 2010). Similar findings revealed that Ugandan parents often delayed testing and disclosure to children because they lacked skills and guidance on how to approach such a sensitive topic (Rwemisisi & Wolff 2008).

A number of studies reported on the use of deception in disclosing HIV diagnosis to children which are in accord with the findings of this study (Funck-Brentano et al. 1997; Oberdorfer et al. 2006). A study by DeMatteo et al. (2002) found that HIV-positive adults were more likely than those who were negative to feel they were the best person to disclose as they felt they could easily relate with the child’s HIV infection. The literature indicates that caregivers would like the doctor or nurse to conduct disclosure preparation sessions prior to the disclosure and they wished the preparations would include talking about responses to questions the child might ask during disclosure which the caregiver might not have knowledge of (Vaz et al. 2008). Incidents were reported where PLWHA not only had to deal with stigma in their communities, but also in their families. Reactions such as separation of clothing and eating utensils and being excluded from community activities were very common, and these reactions were also identified in this study (Liamputtong, Haritavorn & Kiatying-Angsulee 2009).

### Subtheme four: Negative attitudes from family members and the community (stigmatisation)

Findings of this study also indicated that family members of caregivers with negative attitudes towards children on ARV have insensitive views regarding ways of isolating children on ARV. Some of these included distancing themselves from these children by not sharing with them kitchen utensils, toiletries and sometimes it reaches the level where these children are moved out of their households. The majority of caregivers have reported some of the causes of these reactions or negative attitudes as fear of being infected by HIV. Other caregivers reported some level of stigmatisation of children on ARV medication by family members, especially the husbands or in-laws of the caregivers. Many caregivers seemed to have given up seeking support from government and community structures. Family and community members’ attitudes towards PLWHA were mentioned by some caregivers and may impact on disclosure of the nature of the disease and treatment itself. Family and community members may gossip and criticise PLWHA, uttering verbal abuse against them:

‘I kept giving ARV to my child as a secret. I don’t want to disclose this to the rest of the people because they are talkative. My younger sister was gossiping and criticising my HIV-positive man friend in my presence. I thought that if she is talking about this it means she is also going to talk negatively about my status, in my absentia.’ (Caregiver 005, 45 years old, unmarried, unemployed on CSG)

Caregivers decide to hide their caring roles because of their imaginary thoughts of suffering from gossip, criticisms and verbal abuse against them after disclosing their responsibilities towards children on ART:

‘His classmates said the child is suffering from a big disease, child came home crying.’ (Caregiver 008, 73 years old, receiving CSG and also OAP)

Research conducted by Pallangyo and Mayers (2009) concurs with the findings of this study where it is indicated that people experience care of HIV-positive children differently which most of the time is negative; however, it was identified that caregivers do continue to provide the care needed. The community and neighbours appreciate what the caregivers are doing, that is, caring for HIV orphans, but they do not want to be physically involved (Gumede 2003).

‘I was seriously hurt when the child was seriously ill, I was isolated, and nobody wanted to come closer to me.’ (Caregiver 005, 45 years old, unmarried, unemployed on CSG)

The study conducted in Accra, capital city of Ghana, revealed that caregivers had gone to great lengths to ‘hide’ not only their patients, but also their caregiving activities, resulting in the social isolation of both patients and their caregivers. In this study, some of the caregivers live in secrecy, not sharing their family member’s diagnosis with extended family members. Stigma resulted in negative attitudes of neighbours, relatives and healthcare workers towards caregivers and their patients (Demmer 2011; Mwinituo & Mill 2006).

### Subtheme five: Learning disabilities

Some caregivers mentioned ARV medication as a contributing factor of learning disabilities. In this study, the findings revealed that caregivers were compelled to seek intervention from a psychologist because of the child’s poor school progress. It was revealed from data that some of the caregivers attributed poor school progress to absenteeism as children were said to be absent when they go for check-ups or when they are ill and sometimes admitted to hospital.

‘At the age of 11 years, my child is not progressing well at school. He is unable to write the word mama. His marks range from 1 to 4 only. I have taken him to the psychologist. Sometimes I think it’s because he is not well most of the time. There is a time where he was admitted to hospital for more than a month which makes it difficult for him to catch up.’ (Caregiver 010, single domestic worker, earning R1000 and child receiving CSG)

The literature indicates that caring for children with learning disabilities was perceived as difficult and frustrating, yet rewarding. This difficulty was noted to be compounded by caregivers’ lack of skills and knowledge of caring for these children (Sandy, Kgole & Mavundla 2013). The aetiology is multifactorial and some factors include pathology directly related to damage by the virus, lack of nutrition and lack of efficacy of ARV because of starting late, non-adherence, etc. Thus, interventions are vital to address the issue (Achema & Ncama 2015).

### Subtheme six: Inadequate support services from government and community structures

Participants mentioned that restrictions imposed by the South Africa Social Security Agency (SASSA) and community structures result in inadequate support for caregivers. In this study, classification of children in need of special care by social workers and community structures forced caregivers to lose hope in seeking support services from the government and community structures as these are characterised by favouritism. Some of the caregivers mentioned that they had given up seeking support from community and government structures:

‘Government and community structures do not classify children on child support grant as children in need of special care because they do not give them food parcels. In our community, for orphans to receive food parcels, SASSA department needs a letter from the chief, whereas in certain villages orphans receive food parcels. One time I visited the social worker for food parcels and I was told children on CSG cannot be assisted.’ (Caregiver 012, 65 years old, pensioner, receiving pension grant)

In this study, it was revealed that caregivers are receiving inadequate support from the government and community structures; other caregivers have also lost hope:

‘Grandmother is worried about being inaccessible to the late parent’s finances, as it was indicated that the invested money will be received by the child at maturity level, at the age of eighteen years. Grandmother was referred to social workers, but she is challenged by long distance, no transport costs to deliver immobile grandmother to the social workers. Grandmother is now taking care of an infant.’ (Caregiver 007, 70 years old, depending on CSG and old-age pension fund)

A study in Agincourt, rural SA, showed that knowledge of an HIV-positive status has enhanced caregivers’ competency in providing care to affected children. Many caregivers received support from family members, whereas few had access to special support and services for PLWHA, such as food supplements or support groups, which were related to the stigma associated with HIV. This poor support was said to hinder caregivers from disclosing the child’s status outside family and constraining access to special external support. There was also reluctance to transfer this knowledge to the next line caregiver. The truth, therefore, only came out when the child’s mother died. Prior to that, non-maternal caregivers were unaware of the child’s HIV-positive status (Venkataramani et al. 2009).

Immobility as well as distance to health facilities were alluded to as barriers for guardians who have difficulties in walking. These prevented children from attending their review dates and picking up ARV supplies (Skovdal et al. 2011). The literature indicates that the process of obtaining government services is difficult for orphans, vulnerable children and their guardians when uncooperative officials and bureaucratic systems often hinder the Centre for Positive Care efforts. Potential beneficiaries (orphans, vulnerable children and their guardians) are sometimes arbitrarily placed on waiting lists, even if proper procedure has been followed (Byenkya & Njaramba 2008).

## Limitations of the study

There were some difficulties in recruiting caregivers of children on ARV medication below 2 years of age as the majority of them did not honour their appointments, provided invalid cellphone numbers or were not available on their cellphone for confirming an appointment telephonically. Because a confidante is no longer compulsory, the majority of children above 12 years of age were accompanied by their elder siblings for follow-up care. The study did not include caregivers below the age of 18 years. Immobile elderly caregivers did not avail themselves because of their inability to walk. The study was confined to the primary health centres, and therefore the findings may not be transferable to other settings.

## Recommendations

It is recommended that all caregivers be motivated to participate in community projects to generate income and alleviate poverty. The government should develop organisations or community centres that will build capacity and empower caregivers to cope with challenges encountered during the caring of children on ARV medication through programmes that would:

- Provide workshops on skills training, income generation and food gardening.
- Provide counselling and information dissemination to caregivers and their children to assist them in understanding the available treatment.
- Train caregivers in parenting, trauma counselling and coping skills.
- Child care forums need to be initiated in each drop-in centre in order to address the stigma and discrimination experienced by children on ARV medication. Social workers, psychologists and religious ministers should be available to caregivers and children on ARV medication in need of counselling.

There is a need for greater involvement of men in caregiving roles and the equal sharing of care work between women and men, for example initiatives that increase male participation in providing care for children on ARV medication not only reduces the burden on women, but also results in reducing negative attitudes, stigmatisation and discrimination against children on ARV medication.

Government needs to consider revising criteria with regard to HIV-positive children in food insecure communities for accessing social grants as suggested by caregivers.

There is a need to develop caregiving policies and programmes that are informed by a full agenda on national AIDS strategies. Participation and involvement of primary and secondary caregivers in shaping national policies and solutions need to be developed.

## Conclusion

There is evidence from this study that caregivers are experiencing challenges as they supervise ARV medication adherence among children. The challenges range from stigma to poverty as these interfere with daily meals that are necessary for taking treatment. However, it should be noted that success stories have also been identified, although few, as they are outnumbered by challenges.
